# A Perspective on the Roles of Adjuvants in Developing Highly Potent COVID-19 Vaccines

**DOI:** 10.3390/v14020387

**Published:** 2022-02-14

**Authors:** Naru Zhang, Kangchen Li, Zezhong Liu, Kutty Selva Nandakumar, Shibo Jiang

**Affiliations:** 1 Department of Clinical Medicine, School of Medicine, Zhejiang University City College, Hangzhou 310015, China; 31904208@stu.zucc.edu.cn; 2 Key Laboratory of Medical Molecular Virology (MOE/NHC/CAMS), School of Basic Medical Sciences, Shanghai Institute of Infectious Disease and Biosecurity, Fudan University, Shanghai 200032, China; zzliu17@fudan.edu.cn; 3 Department of Medical Biochemistry and Biophysics, Karolinska Institute, 17177 Stockholm, Sweden; ksnandakumar@outlook.com

**Keywords:** COVID-19, SARS-CoV-2, variants, vaccine, adjuvant

## Abstract

Several countries have made unremitting efforts to develop an optimal vaccine in the fight against coronavirus disease 2019 (COVID-19) caused by severe acute respiratory syndrome coronavirus 2 (SARS-CoV-2). With the increasing occurrence of SARS-CoV-2 variants, current vaccines show decreased neutralizing activities, especially towards the Omicron variant. In this context, adding appropriate adjuvants to COVID-19 vaccines can substantially reduce the number of required doses and improve efficacy or cross-neutralizing protection. We mainly focus on research progress and achievements associated with adjuvanted COVID-19 subunit and inactivated vaccines. We further compare the advantages and disadvantages of different adjuvant formulations in order to provide a scientific reference for designing an effective strategy for future vaccine development.

## 1. Introduction

Coronavirus disease 2019 (COVID-19) is an infectious disease caused by the pathogen of severe acute respiratory syndrome coronavirus 2 (SARS-CoV-2). In December 2019, the first known case was identified in Hubei Province, China [1]. Since then, the disease has spread around the world, resulting in a serious epidemic of unprecedented nature [2]. The cumulative number of confirmed cases worldwide had reached almost 387 million by 4 February 2022 [3], and the COVID-19 pandemic is expected to result in a USD 10 trillion economic loss to the world [4], apart from the human loss and pain. Potential treatment options available for targeting SARS-CoV-2 were reviewed elsewhere [5]. Here, we mainly discuss improving COVID-19 vaccines with the aim of establishing an acquired immunity against SARS-CoV-2 and its variants using different adjuvants. Many COVID-19 vaccines are widely accepted for their role in reducing transmission, severity and mortality of the pneumonia virus [6]. According to an official report of the World Health Organization, as of 1 February 2022, a total of 10 billion doses of different forms of COVID-19 vaccines had been administered worldwide [3].

SARS-CoV-2 encodes 15 non-structural proteins, 4 major structural proteins (spike (S), membrane (M), envelope (E) and the nucleocapsid (N) protein), and 8 auxiliary proteins [7]. Most clinical trial platforms for vaccine candidates have focused on the S protein and its variants [8], because the S protein affects viral fusion and cell entry. The receptor-binding domain (RBD) of the S protein is the main target of neutralizing antibodies [9]. At this point in time, the currently approved COVID-19 vaccines, such as NVX-CoV2373 and EpiVacCorona (S protein-based vaccines), CoronaVac and BBIBP-CorV (inactivated vaccines), have shown decreased protection against variants of concern (VOCs) of SARS-CoV-2, and there have been increasing numbers of breakthrough infections with VOCs in the vaccinated population [10]. This calls for stepped-up development of adjuvanted vaccines able to induce an effective and durable protective immunity against SARS-CoV-2 and its emerging variants. Adjuvants are critical components for both subunit vaccines and certain inactivated vaccines [11]. Multimeric display of the antigens in combination with a powerful adjuvant could enhance the longevity and potency of the induced immune responses in the host.

Adjuvants are commonly used to modulate or enhance the effectiveness of a vaccine by mimicking specific conserved molecules called pathogen-associated molecular patterns (PAMPs). For example, liposomes, lipopolysaccharides, components of bacterial cell walls, and endocytosed nucleic acids such as RNA, dsRNA, and ssDNA stimulate and activate the immune system for a better response. The immune system has evolved to recognize these specific antigenic determinants using germline-encoded pattern recognition receptors (PRRs) expressed by dendritic cells (DCs), granulocytes and epithelial cells. Vaccine adjuvants can stimulate DCs, lymphocytes and macrophages by imitating natural infection and, consequently, increase innate immune responses by several fold. Different kinds of adjuvants are available, such as aluminum (or alum)-based adjuvants, stimulator of interferon gene (STING) agonist-based adjuvants, emulsion adjuvants, and toll-like receptor (TLR) agonist adjuvants. All of these can be used to design an effective vaccine formulation [12,13,14]. Current progress in vaccine research with different adjuvant formulations may provide key information for later clinical trials and help in the selection of adjuvants with superior efficacy and safety. Therefore, we focus on the latest research progress on adjuvants used in COVID-19 vaccines in order to provide a reference point for subsequent vaccine development.

## 2. Adjuvants Used with SARS-CoV-2 Vaccine Candidates

### 2.1. Aluminum-Based Adjuvants

The alum-based adjuvants are considered safe and effective as enhancers for SARS-CoV-2 protein-based subunit vaccines as well as inactivated vaccines, which have been widely investigated in clinical trials [15,16,17]. Antigen internalization does not require co-phagocytosis of alum components. In addition, antigens are processed via the lysosomal pathway and presented along with major histocompatibility complex II (MHC-II), rather than cross-delivery to MHC-I-mediated cellular immunity. However, poor cellular immune response, along with a predominance of T helper 2 (Th2) cell response, restricts the use of alum as an adjuvant for many candidate antigens. In order to solve this problem, alum is packaged on the squalene/water interface to form an alum-stabilized Pickering emulsion (PAPE) [18]. PAPE is based on clinically approved alum and squalene, and exhibits a good biosafety profile. It also has a higher affinity for uptake by DCs, which leads to an efficient delivery of antigens and an enhanced cross presentation. This results in strong humoral and cellular immune responses [19]. PAPE may provide potential insights into a safe and effective COVID-19 vaccine adjuvant platform. Studies have shown that mice co-administered with a pharmacological inhibitor of elastase and alum-adsorbed SARS-CoV-2 S protein-based vaccines can develop high-affinity serum IgG and IgA antibodies [19], indicating that suppression of neutrophil elastase could improve the efficacy of alum-based injected vaccines. Immunization with the inactivated vaccine candidate BIV1-CovIran formulated with alum adjuvant elicited a high level of SARS-CoV-2-specific neutralizing antibodies in mice, rabbits and nonhuman primates (NHPs) [20]. Immunization with two doses of psoralen-inactivated whole-SARS-CoV-2 vaccine (SARS-CoV-2 PsIV) formulated with alum adjuvant generated dose-dependent neutralizing antibodies and Th2 cell responses in mice [21]. A double-blind, randomized, and controlled phase 1/2 clinical trial was conducted to evaluate the safety, tolerability and immunogenicity of the inactivated SARS-CoV-2 vaccine CoronaVac formulated with alum adjuvant in healthy children and adolescents aged 3–17 years, and an alum-only group was included as a control group. Results suggested that CoronaVac was well tolerated, safe, and induced humoral responses with no detectable antibody responses in the alum-only group [16]. Another double-blind, placebo-controlled phase 1/2 clinical trial was conducted to evaluate the safety and immunogenicity of an inactivated SARS-CoV-2 vaccine formulated with or without alum adjuvant in Chinese adults aged ≥18 years. The results suggested that the inactivated vaccine was well tolerated and immunogenic in both younger and older adults with no detectable antibody responses in all placebo groups [22]. The vaccine adjuvanted with alum alone was compared with the yeast-expressed trimeric form of the RBD protein adjuvanted with a TLR7/8 agonist and alum formulation, termed as alum-3M-052. The latter formulation induced 100-fold higher neutralizing antibodies, improved Th1-based CD4^+^ T cell responses, and increased CD8^+^ T cell reactions [23,24], thus making it a powerful and scalable COVID-19 vaccine candidate (Table 1). 

Use of alum adjuvants has raised concerns that Th2 immune responses might promote vaccine-enhanced respiratory disease (VAERD). However, no evidence of this has been found in the current studies undertaken with alum adjuvant added to the coronavirus vaccines [25]. Another study claimed that the addition of alum adjuvant in vaccines potentially led to autism, or other chronic diseases, but this has since been thoroughly disproved [26].

### 2.2. STING Agonist-Based Adjuvants

The cyclic GMP-AMP synthase-stimulator of interferon genes (cGAS-STING) pathway has gained much attention in studies related to innate immunity. A STING agonist makes an ideal adjuvant by activating STING to modulate the major immune cell factors and induce robust humoral, as well as cellular, immune responses [13]. Several strategies are employed to activate the cGAS-STING pathway, including small-molecule agonists, polymers and manganese ions. Here, we mainly discuss small-molecule STING agonist- and manganese-based adjuvants (Table 1).

#### 2.2.1. Small-Molecule STING Agonist-Based Adjuvants

CF501, a small-molecule non-nucleotide STING agonist, has been reported recently as a promising adjuvant [27]. CF501 is able to enter the host cells to activate the STING pathway, as demonstrated by high levels of phosphorylated STING, TBK1 and IRF3 in THP-1 cells, and proinflammatory cytokines and type I IFNs in the draining lymph nodes, thus potently activating an innate immune response in vivo [27]. Intramuscular administration of CF501-adjuvanted SARS-CoV-2 RBD-Fc-based vaccine (CF501/RBD-Fc) was compared to alum- and cGAMP-adjuvanted RBD-Fc vaccines. The CF501 formulation elicited significantly stronger humoral and cellular immune responses against SARS-CoV-2 and its variants, SARS-CoV and SARSr-CoVs from bats in different animal models, including mice, rabbits and NHPs without concomitant exacerbated inflammation [27]. Moreover, when immunized with the CF501/RBD-Fc vaccine, human angiotensin-converting enzyme 2 transgenic mice (hACE2-Tg mice) were almost completely protected against SARS-CoV-2 challenge at 6 months post-immunization. Neutralizing antibody titers in the sera of CF501/RBD-Fc-immunized rhesus macaques lasted at high levels for 6 months, which effectively protected these rhesus macaques against SARS-CoV-2 challenge [27]. Since CF501 exhibited excellent adjuvant effects in terms of inducing a potent, broad and long-lasting immune protection, it could be an alternative adjuvant to boost the original subunit vaccines.

#### 2.2.2. Manganese-Based Adjuvants

Manganese is a necessary micronutrient for various biological activities. In host defense, Mn^2+^ is a potent innate immune stimulator that induces the production of type I interferon (IFN) and cytokines in the absence of any infection [28,29]. 

IFN response is the core of host resistance to SARS-CoV-2 and other viral infections. In vitro studies have revealed that SARS-CoV-2 is more susceptible to IFNα and IFNβ therapy than SARS-CoV [45]. However, as a consequence of its neurotoxicity and non-specific distribution, researchers have prepared nano-manganese (nanoMn) based on Mn^2+^ by using chemical engineering technology [30]. Compared with free Mn^2+^, nanoMn strengthens cellular uptake and maintains a sustained release of Mn^2+^, thereby enhancing IFN response and triggering broad-spectrum anti-coronavirus effects [30]. NanoMn is preferentially phagocytosed by macrophages, and it promotes polarization of macrophages to M1-phenotype and recruitment of monocytes to inflammatory lesions, which was ultimately shown to enhance anti-viral immunity and improve coronavirus-induced tissue damage [46]. In addition, as a vaccine adjuvant, nanoMn facilitates antigen presentation, virus-specific memory T cell development and an increased adaptive immunity in hosts. As evidenced from pharmacokinetic and safety assessments, nanoMn treatment, by limiting neuronal manganese accumulation, rarely causes neuroinflammation [46]. Therefore, nanoMn facilitates development of a safe, simple and powerful nanoparticle-based anti-SARS-CoV-2 strategy. A nano-vaccine consisting of the RBD of S protein and a manganese nano-adjuvant (MnARK) induced both humoral and cellular immune responses [31]. Moreover, compared to the alum-adjuvanted RBD vaccine, the MnARK-containing vaccine was superior in neutralizing the virus in vitro, even at a lower antigen dose with fewer injections [31], suggesting that the MnARK adjuvant should be considered for COVID-19 subunit vaccine development. 

### 2.3. Oil-in-Water Emulsion Adjuvants 

Oil-in-water emulsion adjuvants based on biodegradable and biocompatible squalene were first prepared in the 1990s [47]. This adjuvant saw significant progress in vaccine preparations with more than 200 million doses during the H1N1 pandemic in 2009 [48]. This demonstrated safety and efficacy has, once again, placed emulsion adjuvants at the forefront of the current COVID-19 pandemic. For example, a phase I double-blind, placebo-controlled, block-randomized trial in Brisbane, Queensland, Australia, showed that an S glycoprotein-clamp (sclamp) vaccine adjuvanted with MF59 induced a strong neutralizing immune response and stimulated S protein-specific T cell responses. This was speculated to have a protective effect against SARS-CoV-2 infection [32]. Researchers have combined the soluble prefusion-stabilized S trimers (preS dTM) from SARS-CoV-2 with the adjuvant AS03 and injected it twice into NHPs. Results suggest that the IgG antibodies induced by this adjuvanted vaccine can protect NHPs against SARS-CoV-2 infection [33]. Similarly, interim results from a randomized, placebo-controlled phase 1–2 dose-range study in healthy adults demonstrated that the preS dTM vaccine adjuvanted with AS03 has effectively promoted the production of anti-virus neutralizing antibodies [34]. However, both optimal antigen formulation and dosage for these mixed vaccine candidates adjuvanted with AS03 require further study.

The use of an oil-in-water emulsion adjuvant could significantly reduce the dose of the antigen and enhance the production of the antigen-specific antibodies [49], allowing more people to receive the vaccinations and accelerating the establishment of herd immunity [49]. In addition, since the manufacturing facilities for emulsion adjuvants are already in place, millions of doses could be quickly prepared and supplied for later trials and licensed use. At the same time, however, emulsion adjuvants have some limitations. For instance, antigens, especially key structural epitopes, are more unstable in the presence of surfactants and at the interface of emulsion adjuvants. Nonetheless, in the context of the current pandemic, separate multi-dose antigens and an adjuvant may still be the preferred global approach to optimize the available resources to enhance the accessibility to potential vaccines (Table 1). 

### 2.4. TLR Agonist Adjuvants

TLRs are key innate immune receptors. They activate the production of downstream IFN, as well as proinflammatory cytokines and chemokines, by recognizing PAMPs through several different signaling pathways. This limits infection and promotes adaptive immune responses [35]. However, the activation of TLRs may be a double-edged sword in that overactivation of TLR signaling pathways may lead to immune-mediated pathological responses, rather than protection [50]. This is particularly evident in the case of SARS-CoV-2 infection [51]. For example, single-stranded RNA (ssRNA) fragments from the SARS-CoV-2 genome could directly activate endosomal TLR7/8 and MyD88 pathway to induce lung inflammation [52]. These findings may also suggest that TLR7/8 agonists, if acting as adjuvants, may induce potent innate immune responses via an MyD88-dependent mechanism. Therefore, researchers should continue to investigate the role of TLRs in SARS-CoV-2 infection and immune pathogenesis. Such mechanistic understanding is key to formulating strategies to treat and prevent COVID-19, as well as design optimal TLR agonists as COVID-19 vaccine adjuvants. Selective targeting of TLRs using high-affinity small molecules could be one such approach [53]. 

An inactivated rabies-vectored SARS-CoV-2 S1 vaccine called CORAVAX was adjuvanted with MPLA-AddaVax, a TLR4 agonist. It was shown to induce high levels of neutralizing antibodies and a strong Th1-biased immune response. As a result, Syrian hamsters were protected from weight loss and viral replication in the lungs and nasal turbinates 3 days after SARS-CoV-2 challenge [36]. In the ferret model, SARS-CoV-2 antigens with TLR1/2 and TLR3 agonists (L-pampo) induced strong humoral and cellular immune responses, which significantly reduced the viral load in their nasal wash [37]. Pam3CSK4 is a TLR1/2 agonist and Pam3CSK4-RBD is a vaccine candidate in which the N-terminal of RBD was site-selectively oxidized by transmission and conjugated with Pam3CSK 4. It induced potent RBD-specific antibodies inhibiting the binding of RBD to ACE2 and protected cells from infection by SARS-CoV-2 and four VOCs (Alpha, Beta, Gamma and Delta). Pam3CSK4 adjuvant also enhanced RBD-specific antibodies and cellular immune responses [38]. Imidazole scaffold-based compounds are widely used in the development of therapeutic drugs [54]. Recently, a novel amphiphilic imidazoquinoline (IMDQ-PEG-CHOL) TLR7/8 adjuvant consisting of an imidazoquinoline conjugated to the chain end of a cholesterol-poly (ethylene glycol) macromolecular amphiphile was developed. It was shown to induce a protective immune response against SARS-CoV-2 after a single vaccination with trimeric recombinant SARS-CoV-2 S protein in the BALB/c mouse model [39]. IMDQ-PEG-CHOL-adjuvanted vaccine also induced enhanced ELISA and in vitro microneutralization antibody titers. This vaccine led to a more balanced IgG2a/IgG1 response, which controlled SARS-CoV-2 replication in mouse lungs [39]. Collectively, these results suggest that IMDQ-PEG-CHOL could be a promising adjuvant against SARS-CoV-2 infection. A whole inactivated SARS-CoV-2 vaccine, termed BBV152, was formulated with a TLR7/8 agonist molecule adsorbed to alum (Algel-IMDG). Its clinical efficacy was evaluated in a randomized, double-blind, placebo-controlled phase 3 clinical trial in 25 Indian hospitals/medical clinics. Results showed that the BBV152 vaccine was highly efficacious against laboratory-confirmed symptomatic COVID-19 disease in adults with no safety concerns raised in this interim analysis [40]. It is well known that an appropriate level of IFN production and an effective control of inflammation are imperative to reduce the incidence and severity of COVID-19 caused by overproduction of cytokines, which can be achieved by modulating TLR-mediated immune responses [55]. However, until now, clinical use of COVID-19 vaccine with TLR agonists as an adjuvant has not been reported. Thus, more extensive research is urgently needed in this area (Table 1).

### 2.5. Other Adjuvants

Other types of adjuvants have also been used in COVID-19 vaccine-related studies. With their more adaptable characteristics, cationic nanocarriers such as polyethyleneimine (PEI), N-[1-(2,3-Dioleoyloxy) propyl]-*N*,*N*,*N*-trimethylammonium chloride (DOTAP), and chitosan are safe and easy to prepare. Accordingly, they have a wide range of application prospects, and they can be specially customized according to the requirements of different vaccines [41]. Lei et al. have investigated the above three mentioned cationic nanocarriers and showed that they are effective adjuvants for mucosal and intramuscular immunization with SARS-CoV-2 recombinant RBD vaccine. Compared with DOTAP and chitosan, PEI is the most potent adjuvant to enhance the humoral and cellular immune responses [41]. Jeffrey and colleagues developed another S protein-based vaccine platform called Man-TLR7. They found that this polymeric glycol-adjuvant improved the immunogenicity of the S protein by inducing a powerful neutralizing humoral immune response and high-quality cellular immune responses [56]. Pamela’s team invented an intranasal vaccine candidate, which formulated S protein with a nanoemulsion (NE) that activated NLRP3 and TLRs with the RNA agonist RIG-I (IVT DI). Passive transfer of vaccinated mouse sera was shown to protect naïve C57BL/6 mice against both homologous SARS-CoV-2 and a variant harboring the N501Y mutation shared by B1.1.7 and B.1.351, as well as challenge by P.1 variants [57]. As a result, a valid cross-protection was established against SARS-CoV-2 drift variants. The use of a GM-CSF adjuvant enhanced a gamma-irradiated inactivated vaccine to induce significantly higher neutralizing antibodies in the absence of antibody-dependent enhancement [58]. An intranasally administered candidate vaccine was developed by combining the N protein and prefusion-full S protein (SFL_mut_) with two adjuvants of flagellin (KF) and cyclic GMP-AMP (cGAMP). This formulation elicited a strong systemic and mucosal humoral immunity, which protected against lethal SARS-CoV-2 challenge with superior protection in the upper respiratory tract compared with lethally challenged mice immunized with an inactivated vaccine [59]. Clinical trials of NVX-CoV2737, which is formulated with the trimeric full-length SARS-CoV-2 S protein and Matrix-M1 adjuvant, have shown that this Matrix-M1-adjuvanted protein vaccine elicits CD4+ T-cell responses biased toward a Th1 phenotype [42,43]. The SARS-CoV-2 S protein formulated with Advax-SM, a combination adjuvant consisting of delta inulin polysaccharide particles (Advax™) and a TLR9-active oligonucleotide-CpG55.2, induced neutralizing antibodies in mice that could neutralize infection by the SARS-CoV-2 wildtype lineage B.1.319 and cross-neutralize the VOC Alpha (B.1.1.7), and protected ferrets against SARS-CoV-2 infection [44].

## 3. Conclusions and Future Perspectives

Alum is still the dominant adjuvant in most subunit and inactivated vaccines. Although it has been safely used in clinics for many years, we still have to pay attention to the potential risk of autoimmune/inflammatory adverse events following injection of alum-adjuvanted vaccines [60] or the vaccine-associated disease enhancement [61]. Comprehensive safety evaluation is essential in the development of COVID-19 vaccines. Alum adjuvants are considered to activate the pro-inflammatory NLPR3 pathway and stimulate preferentially prime Th2-type cell responses. To address this, scientists have combined alum with PRR-based adjuvants, such as TLR agonists, in order to synergistically enhance the immunogenicity of a vaccine antigen to elicit Th1/Th2 balanced and durable immune responses against infections of viruses, such as SARS-CoV-2 and its VOCs. Emulsion adjuvants, such as MF59 and AS03, have demonstrated good safety and tolerability in the prevention and treatment of COVID-19. The use of an emulsion adjuvant can conserve vaccine doses with the effect of expanding vaccination coverage to attain herd immunity in the fight against COVID-19. However, an antigen could be unstable in the surfactant and at the interface of emulsion adjuvant; therefore, further studies are required. TLR agonist adjuvants have a clear immunomodulatory profile and an excellent ability to regulate IFN production. In addition, nasal mucosal epithelial cells can recognize and take up pathogenic species and/or antigenic components by non-specific endocytosis or by communicating with TLRs [62], which might make TLR agonists appropriate adjuvants for intranasal injection of next-generation COVID-19 vaccines. Nevertheless, to date, no clinical trials with TLR agonist adjuvants have been reported. Therefore, animal model experiments must be performed to give valuable reference points. Thanks to nanotechnology, neurotoxicity of the novel manganese adjuvant was reduced while retaining its ability to activate a stable acquired immunity and elicit a broad spectrum of anti-viral activity. Intramuscular injection of the small-molecule STING agonist-based adjuvants, such as CF501, exhibited excellent adjuvant effects in terms of potency as well as providing broad and long-lasting immune protection [27], suggesting their potential as an injectable adjuvant for further clinical use.

Various adjuvants focus on different targets and properties, thus enabling a wide variety of technical applications to broaden vaccine development. Therefore, it is plausible that more safe and effective adjuvants, or adjuvants in combination with nano-microparticles as appropriate carriers, can be introduced and developed in next-generation vaccines to curb the further spread of COVID-19.

## Figures and Tables

**Table 1:** Adjuvants’ mechanism of action, strategies for enhanced improvement and their status of clinical use.

Adjuvants	Exampled Adjuvants	Vaccine Platform	Administration Route	Action Mechanism	Strategies for Improvement	Ref.
Aluminum hydroxide -based adjuvants	Alhydrogel adjuvant 2% (Aluminum hydroxide gel)	Inactivated vaccines (BIV1-CovIran; psoralen-inactivated whole-SARS-CoV-2; CoronaVac) /Subunit vaccines (trimeric RBD protein)	i.m.; i.d.	Activate pro-inflammatory NLPR3 pathway and stimulate preferentially prime Th2-type cell response	Package alum on the squalene/water interface to form an alum-stabilized PAPE; make nanoparticles of alum	[16,18] [20,21] [22,23,24]
Small molecule STING agonist	CF501	Subunit vaccines (RBD-Fc)	i.m.	Activate STING to modulate major immune cell factors and induce humoral and cellular immune responses	Design appropriate derivatives with balanced potency, good solubility and few side effects	[13,27]
Manganese-based adjuvants	Nano-manganese	Subunit vaccines (RBD protein)	i.m.	Enhance cGAMP production via cGAS activation and increase the binding of cGAMP with STING	Make nanoMn based on Mn^2+^ by using chemical engineering technology	[28,29,30,31]
Oil-in water-based emulsion adjuvants	MF59; AS03	Subunit vaccines (recombinant S protein)	i.m.	Lead to ATP-release in muscle cells and subsequent DC recruitment, CD4^+^ T cell priming and humoral responses	Add appropriate surfactant to make oil droplets homogeneously dispersed throughout the outer water phase	[32,33,34]
TLR agonist adjuvants	LR1/2 and TLR3 agonists; TLR4; TLR7/8 agonists	Subunit vaccines (RBD, RBD-Fc, S1, S proteins) /Inactivated vaccines (BBV152)	i.m.	Activate the production of downstream IFN, pro-inflammatory cytokines and chemokines leading to adaptive immune responses by recognizing the PAMPs through several different signaling pathways	NA	[35,36,37,38,39,40]
Cationic nanocarriers	PEI; DOTAP; Chitosan	Subunit vaccines (RBD protein)	i.m.; i.n.	Activate cytotoxic CD8+ T lymphocytes and CD4+ T helper arm. Enhance the antigen uptake capability of DCs	NA	[41]
Matrix-M1	N/A	Subunit vaccines (trimeric S protein)	i.m.	Stimulate humoral and cellular immune responses to vaccines by inducing CD4+ T-cell responses biased toward a Th1 phenotype	NA	[42,43]
Advax-SM	Delta inulin polysaccharide particles (Advax™) and a TLR9-active oligonucleotide, CpG55.2	Subunit vaccines (ECD of the S protein)	i.m.	Activate CD8+ dendritic cells and induce effective dendritic cell cross-presentation of S protein to CD8+ T cells. Impart a strong Th1 bias and robust T cell responses	NA	[44]

Abbreviations: NLPR3: NOD-like protein receptor 3; Th2: T helper 2; PAPE: Pickering emulsion; STING: stimulator of interferon genes; cGAMP: 2′,3′-cyclic guanosine monophosphate-adenosine monophosphate; cGAS: cyclic guanosine monophosphate (GMP)-adenosine monophosphate (AMP) synthase; IFN: type I interferon; nanoMn: nano-manganese; TLR: toll-like receptor; PAMPs: pathogen-associated molecular patterns; PEI: polyethyleneimine; DOTAP: N-[1-(2,3-Dioleoyloxy) propyl]-*N*,*N*,*N*-trimethylammonium chloride; ECD: extracellular domain; NA, not available.

## Data Availability

Not applicable.
